# Antioxidant, Antibacterial, and Anticancer Activity of Ultrasonic Nanoemulsion of *Cinnamomum Cassia* L. Essential Oil

**DOI:** 10.3390/plants12040834

**Published:** 2023-02-13

**Authors:** Aftab Alam, Mohammad Javed Ansari, Mohammed H. Alqarni, Mohammad Ayman Salkini, Mohammad Raish

**Affiliations:** 1 Department of Pharmacognosy, College of Pharmacy, Prince Sattam Bin Abdulaziz University, Al-Kharj 11942, Saudi Arabia; 2 Department of Pharmaceutics, College of Pharmacy, Prince Sattam Bin Abdulaziz University, Al-Kharj 11942, Saudi Arabia; 3 Department of Pharmaceutics, College of Pharmacy, King Saud University, Riyadh 11451, Saudi Arabia

**Keywords:** *Cinnamomum cassia*, essential oil, antioxidant, nanoemulsion, biological properties

## Abstract

*Cinnamomum cassia* (*C. assia*) has long been used in traditional holistic medicine for its medicinal properties. It is used as an antioxidant, antibacterial, anti-inflammatory and anticancer agent. Cinnamon, in particular, the essential oil of *C. cassia*, has significant biological properties. Despite this, the volatility, stability, and insolubility of *C. cassia* essential oil (CEO) remain the main disadvantages that limit its application, ultimately affecting its pharmacological efficacy. To find a solution to this problem, we developed the CEO nanoemulsion (CEO-NE). For lipophilic compounds, insoluble nanoemulsion-based formulations are a popular delivery strategy. In this research work, a highly stable dosage form named CEO-NE was successfully developed using polysorbate 80 and water. The findings show that the synthesized CEO has a uniform shape with a PDI of 0.380 and an adequate particle size of 221.8 nm. The antioxidant outcomes show excellent results for CEO-NE compared to CEO against DPPH and hydrogen peroxide. The obtained antibacterial activity of CEO-NE was more efficient than that of CEO against *Klebsiella pneumonia* (MTCC 8911) with 0.025% and 0.05%, respectively. The CEO-NE preparation was tested against an alveolar lung adenocarcinoma cell line (A549) with an IC_50_ of 50.21 µg/mL for CEO and 18.05 µg/mL for CEO-NE, respectively. These results are encouraging for future translational studies on CEO-NE use in lung cancer therapy due to its excellent antioxidant, antibacterial, and killing kinetic properties.

## 1. Introduction

Essential oils (EOs) are volatile, aromatic liquids generated by any parts of plants, such as foliage, bark, wood, fruit, seeds, or rhizomes [1]. EOs are the most important raw materials for industries such as perfume, food, and medicine. They have nutritional value and can be used as edible oils [2]. Essential oils are used to enhance the taste and aroma of food. Essential oils are often used as flavor enhancers. They also have a therapeutic purpose in certain cases and possess significant biological activity, which has been extensively documented in recent years [3]. They have been reported to show antibacterial, antiviral, nematicidal, antifungal, insecticidal, and antioxidant properties; essential oils also offer a “green” option in nutrition, medicine, and agriculture [4]. 

In this regard, cinnamon essential oil (CEO), one of the prominent sources of natural EO cinnamaldehyde, has good antimicrobial and antioxidant potential, extensively used as a natural food flavor. The aromatic herb *Cinnamomum cassia* (L.), popularly recognized as “Chinese cinnamon”, belongings to the family of Lauraceae [5]. Essential oils extracted from cinnamon plants comprise primary active metabolites such as cis/trans cinnamaldehyde and trans-anethole, a common bioactive component in the essential oil of cinnamon plants [6]. Extensive chemical and biological analyses of CEO have revealed the presence of other numerous therapeutically active compounds, including cinnamic acid, coumarin, cinnamaldehyde, γ-muurolene, γ-amorphene, α-cubebene, δ-cadinene, (−)-calamenene, and polyphenols [7]. For this reason, CEO is an important raw material in the pharmaceutical industry, used for food product development, pharmaceuticals, and dental products. CEO has attracted much interest in the scientific community because it is utilized in indigenous herbal therapeutics to treat various conditions, including diabetes, ovarian cysts, stomach cramps, kidney ailments, hypertension, and menstrual cramps [8,9,10,11,12].

Unfortunately, due to their poor solubility in water, the effectiveness of essential oil therapy has been severely limited [13]. Apart from this, there are still some limitations, including the fact that CEO is generally sensitive to external factors such as temperature, light, and oxygen. This affects its shelf life and significantly reduces the efficiency of its functional activity, limiting its wide applicability [14]. The most important technique for making EO compatible with aqueous formulations and reducing evaporative losses is encapsulating the essential oils in various nano- and microscale forms [15]. To overcome such obstacles, a good approach is to preserve the properties of CEO and improve its water solubility by encapsulating nanoparticles. In this regard, an oil–water nanoformulation is a good solution. For example, microcapsules containing the essential oils of *Rimulus cinnamon* and *Angelica sinensis* were prepared and evaluated by Lai et al. After 6 months, starch sodium octenyl succinate (SSOS) and maltodextrin (MD) encapsulated EOs showed significantly improved stability, encapsulation efficiency, and drug loading of EOs [16].

Similarly, Liang et al. recently developed characterized CCEO nanoemulsion (CCEO-NE). CCEO-NE has a sphere feature, smooth surface, uniform shape, and average particle size of 221.8 ± 1.95 nm. This experiment found that CCEO remained intact in the intestine, especially in the stomach and intestine. Using whey protein as a packaging technique for CCEO can potentially increase its efficacy, stability, and bioavailability in several contexts [13]. 

Several nanoencapsulation methods have been developed, but encapsulation in nanoparticles or nanoemulsions seems to be the most suitable and promising. In this context, nanoemulsions are described as kinetic stability systems, of which drops generally present a range of sizes from 50 to 200 nm [17]. In addition, depending on the EO, the formation of stable nanoemulsions may depend on the mixing of the EO and the surfactant and/or their ratio, and it may also expect a higher energy input, such as ultrasonic treatment. In this respect, initial efforts focused on the formation of emulsions with food-grade surfactants such as Tween and Span in *Lippia citriodora* [18], *Mentha spicata* [19], *Myrtus communis* [20], and so on.

As a result of these factors, emulsification of CEO has been proposed as a strategy to increase its utility and expand its use in therapeutic formulations. Therefore, this study aimed to prepare nanoemulsions encapsulated with CEO to achieve excellent antimicrobial and anticancer activity using a probe sonicator. In the current research, we developed CEO nanoemulsions (CEO-NE) and further evaluated them for morphology, particle size (Z-average), particle size dispersion index (PDI), zeta potential, encapsulation, and turbidity. After this, they were further evaluated for antioxidant and antibacterial ability against *klebsiella pneumonia* (MTCC 8911) and the human alveolar lung adenocarcinoma cell line (A549) using the assay MTT method. 

## 2. Results

### 2.1. Evaluation of Developed Nanoemulsion

#### 2.1.1. Size and Zeta Potential Analysis of CEO-Loaded Nanoemulsion via Dynamic Light Scattering (DLS) Study

**Figure 1:**
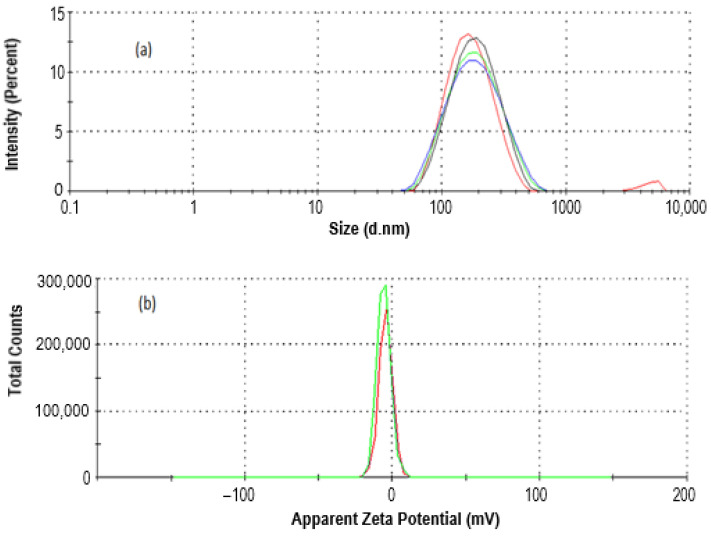
Size and zeta potential analysis of CEO-NE.

Through the use of homogenization technology, we were able to prepare CEO-NE, which resulted in a more stable emulsion by lowering the interfacial tension that exists between the oil phase and the water phase. The average hydrodynamic diameter of the CEO-loaded nanoemulsion was 221.8 ± 11.95 nm with a PDI of 0.38 ± 0.019, as shown in Figure 1a. The physical stability of the CEO-NE was improved, and the particles were homogeneous, which allowed for a lower PDI. The PDI was below 0.7, which is ideal because it shows that the distribution of particle sizes falls within a narrow size range [21]. The zeta potential of the loaded nanoemulsions by CEO was only slightly negative at −5.6 mV, as shown in Figure 1b. The zeta potential value indicates sufficient stability of the optimized formulation. According to the scientific literature, a more excellent absolute value of zeta potential suggests a much more stable suspension [22]. It is vital to evaluate the zeta potential of nanoparticles to make accurate predictions about their stability or the possibility of their agglomeration in body fluids. If this is actively encouraged, it is necessary to measure the zeta potential of nanoparticles to predict their stability or the possibility of aggregation in biological fluids. Additionally, it is known that zeta potential influences the transit of nanoparticles through bodily tissues or the body and their subsequent uptake by cells, as well as the stability of body fluids [23]. 

#### 2.1.2. Morphology Study

**Figure 2:**
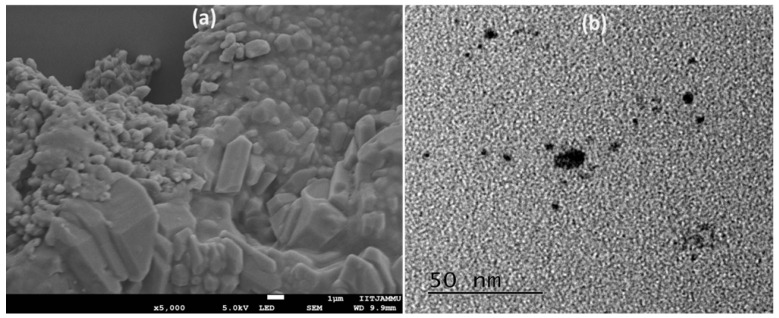
Morphology characterization of nanoemulsion analyzed using (**a**) SEM and (**b**) TEM.

Utilizing a scanning electron microscope, the surface morphology of nanoparticles was analyzed (see Figure 2a). It is more than evident that the nanoemulsion system is homogeneous, the droplet sizes are on the nanoscale, and the droplet shapes are irregular. Moreover, the internal morphology was evaluated via the TEM study. TEM images showed that the sphere of CEO-NE is as shown in Figure 2b. CEO-NE micrographs also demonstrated the formation of spherical nanoparticles in the core–shell. Figure 2b shows several dark areas. This might be because CEO is present in the CEO-NE nuclear envelope. The size determined by TEM does not correspond to the size determined by DLS. The discrepancy in the size distribution may be due to the fact that the two techniques use different principles for the analysis. DLS shows results that include large particle size, as its hydrodynamic diameter is larger than TEM due to a dispersion of electrons in the sample radiation results [24,25]. 

### 2.2. % Entrapment Efficiency (%EE)

The percentage EE of nanoemulsions loaded with CEO-NE was reported to be 63.65 ± 3.182%. This high %EE value suggests that the nanoemulsion system used in this study was successful in encapsulating a large amount of CEO and CEO-NE within the small droplets of the nanoemulsion. This can be attributed to the use of appropriate surfactants and emulsifiers which helped to stabilize the droplets and prevent the active ingredient from leaking out. This result is important for the application of these nanoemulsions as a delivery system for CEO-NE, as it indicates that a high amount of the active ingredient will be available for release and absorption into the skin, leading to an effective and efficient treatment. Additionally, a high EE value also suggests that the nanoemulsion system is stable and will not experience a significant loss in the active ingredient over time.

### 2.3. In Vitro Drug Release and Kinetic Study

**Figure 3:**
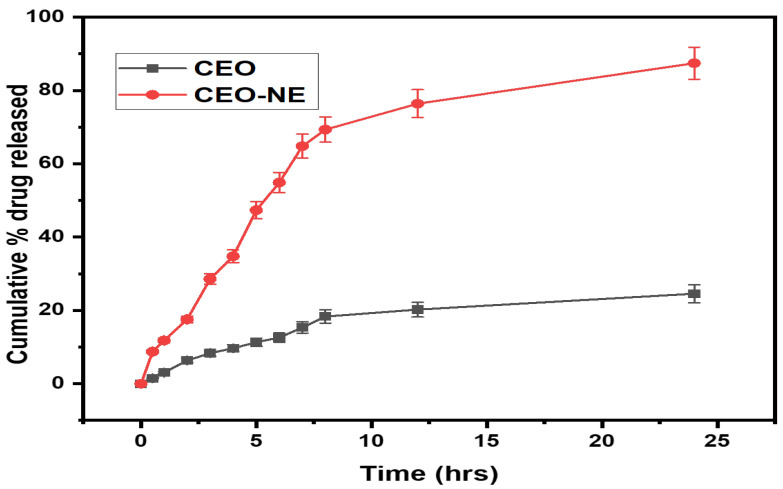
Comparative in vitro drug release study in pH 6.2 PBS solution for 24 h.

**Figure 4:**
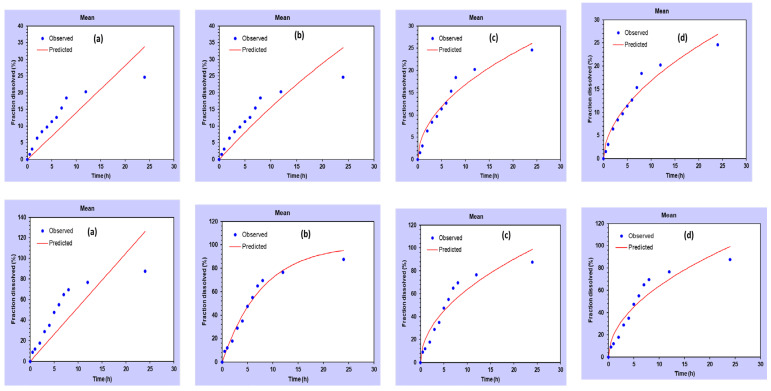
Comparative kinetic data for CEO (upper) and CEO-NE (lower): (**a**) zero order; (**b**) first order; (**c**) Higuchi; (**d**) Kors–Peppas.

The in vitro drug release study, as shown in Figure 3, evaluated the percentage of CEO (drug) release from the nanoemulsion over a 24 h period in a medium containing pH 6.0 PBS buffer. The results indicate that the bare CEO (without nanoemulsion) released significantly less drug, with only 8.32 ± 0.416% released after 3 h, 12.59 ± 0.6295% after 6 h, and 24.56 ± 1.22% after 24 h. In contrast, the CEO-NE (nanoemulsion with CEO) released a much higher amount of drug, with 28.56 ± 1.428% released after 3 h, 54.87 ± 2.743% after 6 h, and 87.42 ± 4.371% after 24 h. This suggests that the use of nanoemulsion significantly improves the release of the drug. Furthermore, the kinetic study (Figure 4 and Table 1) revealed that the CEO-NE formulations followed first-order kinetics, with an r^2^ value of 0.978. This means that the rate of drug release is directly proportional to the amount of drug remaining in the system, indicating a consistent and predictable release of the drug over time. Overall, these results suggest that the use of nanoemulsion technology improves the release of the drug and exhibits consistent and predictable kinetics. This could have potential implications for the development of more effective and efficient drug delivery systems.

The results of this study support the hypothesis that the smaller size of the nanoemulsion particles leads to increased surface area for diffusion and release of active ingredients. Specifically, the nanoemulsion particles showed a significantly higher percentage of drug release compared to the bare CEO particles. This can be attributed to the increased surface area of the nanoemulsion particles, which allows for more efficient diffusion and release of the drug. In addition, the study revealed that the solubility of CEO may have played a role in the drug release results. The bare CEO particles exhibited a very low percentage of drug release, which can be attributed to their low solubility. This shows the importance of considering solubility when developing drug release systems. Furthermore, the study found that the Kors–Peppas model yielded the highest r^2^ value for the CEO nanoemulsion system, indicating that the release of the drug from the nanoemulsion particles follows a non-Fickian diffusion mechanism. This suggests that the release of the drug from the nanoemulsion particles depends not only on the surface area, but also on other factors such as the properties of the carrier and the interactions between the drug and the carrier. In summary, the results of this study show that the use of nanoemulsion particles as a drug delivery system can significantly enhance drug release compared to bare CEO particles. In addition, the study highlights the importance of considering factors such as solubility and release mechanisms when developing drug release systems. 

### 2.4. In-Vitro Anti-Oxidant Activity

Antioxidants are compounds that neutralize reactive species by inhibiting oxidation processes, protecting cells from damage that can result from excess reactive oxygen species (ROS). Plant extracts contain many natural components that can act as antioxidants. These naturally occurring antioxidant chemicals are in high demand because they effectively inhibit the ROS-mediated pathogenic mechanisms of a wide range of pathological conditions, such as carcinogenesis and cardiovascular disease. ROS plays a role in developing these diseases [26].

**Figure 5:**
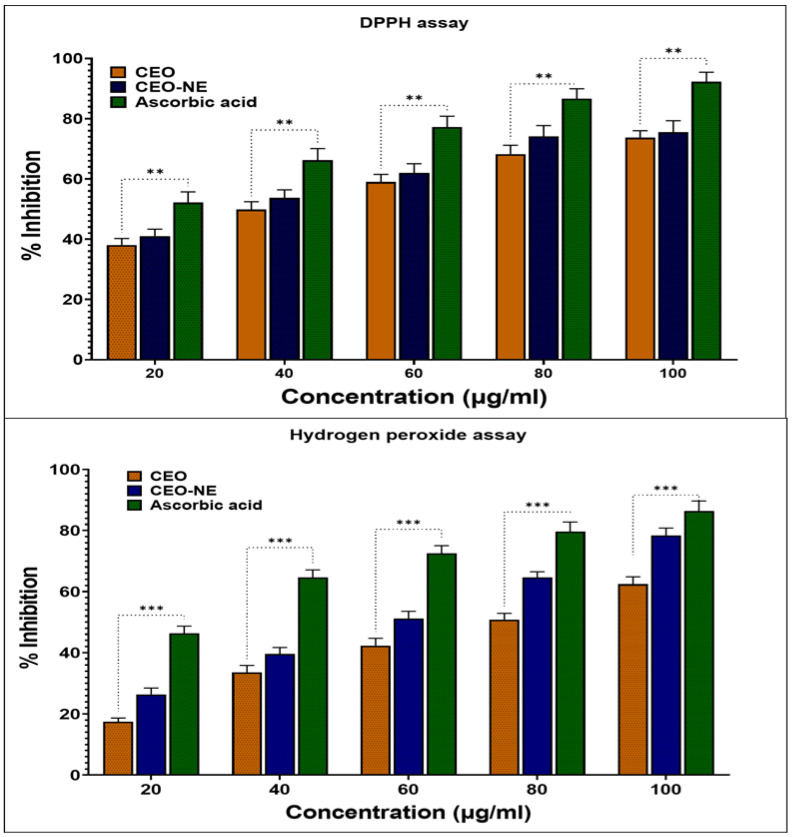
Comparative antioxidant activities: (**A**) DPPH radical scavenging assay of ascorbic acid, CEO, and CEO-NEO; (**B**) hydrogen peroxide assay of ascorbic acid, CEO, and CEO-NEO. *p*-value (** *p* < 0.01 and *** *p* < 0.001), when compared to a standard compound using two-way ANOVA of GraphPad prism 9.5.0.

After the development of the CEO nanocarrier, one of the essential basic investigations is the study of the effectiveness of antioxidants. Antioxidants are effective medicines for a wide range of degenerative conditions. Therefore, the antioxidant capacity of the newly synthesized CEO-NE was investigated using established in vitro techniques. Figure 5 shows a quantitative comparison of the antioxidant capacity of CEO. CEO-NE performed significantly better in the hydrogen peroxide assay and the DPPH radical scavenging experiment than the conventional antioxidant CEO. This was performed after the parameters of the pharmaceutical formulation were determined. As shown in Figure 5, the antioxidant capacity of the biosynthesis CEO-NE was significantly increased in the experiments with five different concentrations. The nanoemulsion without oil did not show antioxidant activity in any in vitro tests. However, the NE nanoparticles synthesized with CEO showed remarkable radical scavenging activity in the hydrogen peroxide and DPPH tests. CEO-NE showed greater antioxidant activity than naked CEO.

In the DPPH assay study, we observed that the IC_50_ value for CEO was 42.65 μg/mL. In contrast, a value of 34.75 μg/mL was reported for CEO-NE, while CEO and CEO-NE trapped hydrogen peroxide assay free radicals at 83.21 µg/mL and 70.89 µg/mL, respectively, as shown in Table 2. It is possible that the encapsulation of CEO in a nanoemulsion carrier is responsible for the enhancement of hydrogen peroxide assessment and DPPH radical scavenging activity. The correlation between the presence of nanoemulsifiers and increased antioxidant activity in natural components is supported by our results, which agree with findings from other studies [27]. The highest antioxidant activity belonged to CEO-NE in the highest concentrations. During the formation of the nanoemulsion, the smaller size of the droplets improves the particular surface area of CEO. This results in free radicals’ rapid and effective adsorption [28]. This result corresponds to our hypothesis that CEO loading increases its antioxidant capacity and inhibits lipid peroxidation within the cell. 

### 2.5. In Vitro Antimicrobial Activity

Using the Gram-negative bacterium *K. pneumonia* (MTCC 8911), the antibacterial activity of CEO and CEO-NE was determined using a well diffusion technique. CEO-NEs showed maximum inhibition against *K. pneumonia* with a MIC of 0.025%, while CEO had a MIC of 0.05%. The MBC was 0.05% and 0.1%. The antimicrobial effect of CEO-NE is not apparent. Nanoparticles’ ability to kill microorganisms varies depending on various factors, including their size, shape, zeta potential, temperature, pH, colloidal state, dose, and variety of microbes. The high antibacterial activity of CEO-NEs can most likely be attributed to their tiny size, relatively low zeta potential (a negative surface charge of −5.00 mV), and the nature of plant phytochemicals in the compound. These findings supported the linear relationship between the concentration of CEO-NEs and the size of the inhibition zone that results [29]. According to Raghupathi and colleagues’ findings, bacteria development can be significantly curbed by using particles with small diameters [30]. Jeong et al. found that the percentage of bacteria killed by a given particle increased the proportion to its size [31].

On the other hand, it is believed that the excessive production of ROS might impair the bacterial defense mechanism by interfering with the normal mitochondrial membrane, the cell membrane, and the organelles beneath it. CEO-NEs have shown a biocidal effect via ROS, which tends to deform bacterial species’ cell walls and membranes, leading to their death [32]. Compared to CEO, the CEO’s antioxidant NEs and antibacterial effects are significantly more robust. According to our research findings, CEO-NE is an effective method of encapsulation that can improve the efficacy, stability, and even bioavailability of CEO-NE in various applications. In addition, CEO-NE has the potential to make CEO-NE more readily available to the body.

### 2.6. Time-Kill Analysis

**Figure 6:**
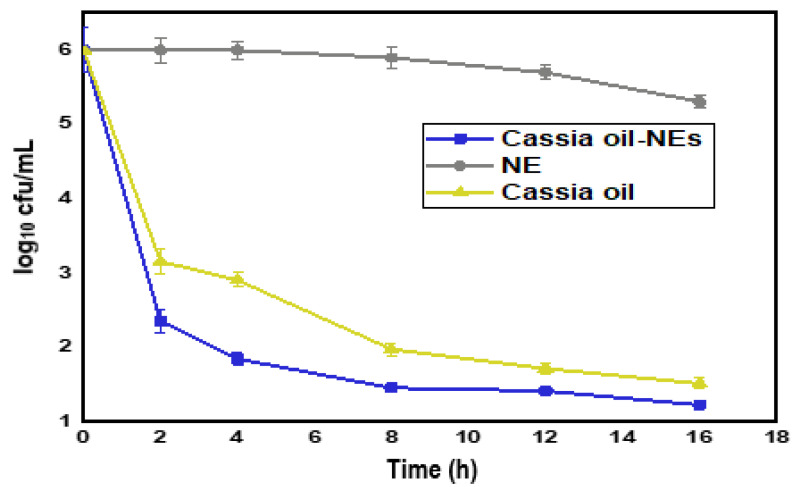
Comparative time-kill analysis of CEO, CEO-NE, and NE.

The bacterial activity of the improved NE formulation against the clinical pathogen *K.pneumonia* was studied. The kinetics of the killing experiment showed the loss of viability as a result of interaction with the synthesized NE; the amount of cells (1 × 10^7^ cfu/mL) was adjusted over time (Figure 6). Immediately after two hours, there was a significant decrease in the number of strains compared to control cells. The initial cell number of bacterial strains was 6.0 (log_10_ cfu/mL), the same as the initial cell number of *K. pneumonia* strains. Subsequently, bacterial cells declined rapidly during the first 8 h of incubation. The result shows a total loss of functionality within 16 h of interaction. All cells were viable, and no cells were killed when they were grown in NE. 

The kinetics of the killed experiment show that both CEO and the CEO-NE have significant antibacterial activity. The high-pressure homogenizer allows the preparation of nanoemulsions with smaller droplets than Ultra-Turrax. The release of volatile compounds from nanoemulsions could be caused by the instability of the interfacial layer of nonionic low-molecular-weight surfactants due to their interaction with the microcell. This is because they tend to bind to certain parts of the biological membrane [33]. Therefore, nanoemulsions would be preferable to bare emulsions because the antimicrobial chemicals would be in more significant contact with the microbial cells due to their smaller particle size, resulting in the faster killing.

### 2.7. Anticancer Activity

**Figure 7:**
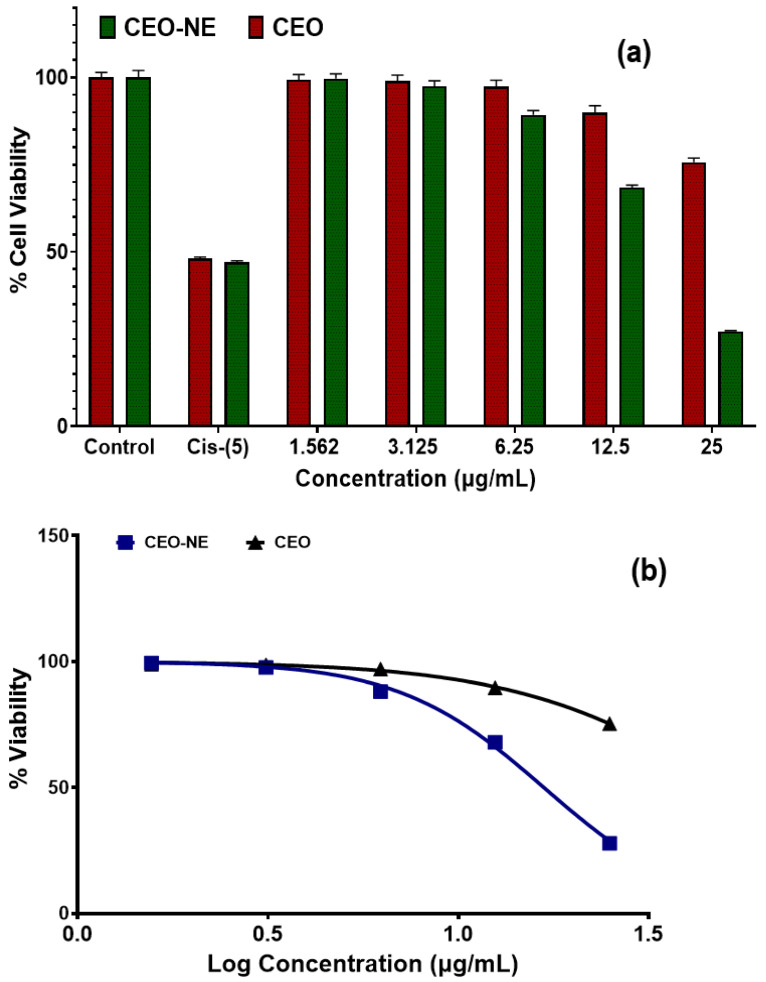
(**a**) Comparative anticancer activity analysis of CEO and CEO-NE; (**b**) % cell-viability versus log concentration (µg/mL) of CEO and CEO-NE against A549 cell lines.

The observations of the cell cytotoxicity study by MTT against the A549 cell lines are shown in Figure 7a,b. The test compounds, cassia oil NE, and cassia oil exhibit significant cytotoxic properties with IC_50_ values of 18.05 µg/mL and 50.21 µg/mL, respectively. The concentration of the drug that must be present to eliminate fifty percent of the cancer cells is the IC_50_ value. The lower this number is, the more cytotoxic the treatment is. Cisplatin (Cis) was used as the standard control in the study; at 5 µg/mL, it was shown to be highly active in comparison to CEONE. On the other hand, test compounds and CEO-NE were shown to be comparatively more effective in comparison to CEO against lung cancer due to their lower IC_50_ values.

### 2.8. Stability Study

The stability of CEO-NE formulations was studied at both 4 °C and room temperature for 0, 15, and 30 days. The particle size, PDI, ZP, and %DEE were evaluated at each time point as given in Table 3. Results showed that the nanoemulsion formulations remained stable at 4 °C with little to no change in particle size, PDI, ZP, and DEE over the course of the study. However, at room temperature, there was an increase in particle size and PDI, as well as a decrease in ZP and DEE over time. These findings indicate that the nanoemulsion formulations are more stable at lower temperatures and suggest that refrigeration should be considered for the storage and transportation of these formulations.

## 3. Discussions

EOs are volatile liquids extracted from the roots, leaves, stems, seeds, flowers, and other parts of aromatic plants. They protect against pathogens and insects. EOs have been extensively studied and used in pharmaceuticals, partly due to their intrinsic pharmacological properties [34]. CEO is a consumable part of foods in the Lauraceae family and has been utilized to treat several ailments [12]. However, the essential oil has been severely restricted because it is hardly soluble in water. Increasing the solubility of the essential oil can be accomplished by using an oil-in-water emulsion, which is an appropriate approach [13].

To realize this objective, the focus of this investigation was on the development and analysis of CEO-NE. It has been reported that the oil-in-water approach can be used to synthesize CEO-NE successfully. Traditional analytical techniques such as DLS, SEM, and TEM can characterize it successfully. The formulation was in the nano-range with a very low PDI and negative zeta potential. The presence of Tween 80 is the primary factor responsible for the negative zeta potential. Tween 80 is a nonionic surfactant with a low molecular weight that rapidly coats newly produced droplets during emulsification. It is preferentially absorbed by the oil surface to produce a neutral or slightly negative electrical charge at the oil–water interface [33]. Therefore, the negative charge of the oil droplets is observed in the present work. Further, the morphology of the newly synthesized CEO was evaluated using SEM and TEM. 

Based on the present study, the conversion of an essential oil (*Cinnamomum cassia*) into a nanoemulsion greatly enhanced its anti-oxidant (DPPH and hydrogen peroxide) and antibacterial activity against *K.pneumonia*. This study observed that CEO nanoemulsion has a better effect on antioxidant and antibacterial activities than pure oil. Thus, our main hypothesis is that CEO NE development increases the pharmacological properties of pure CEO. Furthermore, by studying its bacterial killing kinetics to understand the bactericidal and bacteriostatic effects, CEO-NE showed excellent bactericidal activity against the selected bacterial strains with very slow death kinetics.

We also tested the anticancer activities of CEO-NE and CEO against the A549 cell lines. The IC_50_ values of CEO and the CEO-NE of DPPH free radicals are 31.47 µg/mL and 29.02 µg/mL, respectively. As a result of this, we believe that CEO-NE has the potential to be used as an innovative drug delivery method that is both safe and effective in the treatment of primary lung cancer. There are several reports which confirm the safety of cinnamon essential oil [1,2]. This preliminary study validates using our CEO-NE formulation as an alternative to lung cancer treatment using an inverted pharmacological approach. This study provides evidence of the anti-tumor growth characteristics of CEO-NE for lung cancer. Therefore, CEO-NE therapy can effectively inhibit the proliferation of breast cancer cells by apoptosis. 

## 4. Materials and Methods

CEO was purchased from WinLab, India. Tween 80 and Butanaol were procured from Sigma Aldrich, USA. Other materials such as A549 human alveolar lung adenocarcinoma cell line (NCCS, Pune), DMEM high-glucose media (Cat No: AL007, Himedia, Mumbai, India), Fetal Bovine Serum (#RM10432, Himedia), MTT Reagent (5 mg/mL) (# 4060 Himedia), D-PBS (#TL1006, Himedia), and 96-well plate for culturing the cells (from Corning, NY, USA) were directly procured from the company. There was no additional purification of the solvents and compounds of analytical grade.

### 4.1. Nanoemulsion Preparation of Essential Oils

The CEO-loaded nanoemulsions were prepared using a probe sonicator (VCX 750, SONICS, Connecticut, United States). In short, the oil phase (CEO), the emulsifier (Tween 80), the co-emulsifier (Butanol), and the aqueous phase (distilled water) were added in a proportion of 10%, 40%, 10%, and 40% *v*/*v*, respectively. The resulting mixture was put in an ice bath and subjected to ultrasonication with a 6 mm probe for 3 min with 10 s off/on cycles at 60% amplitude. Nanoemulsions were stored at ambient temperature for their intrinsic stability and observed for phase separation or creaming.

### 4.2. Study of Physical Appearance and Morphology

A scanning electron microscope was utilized to investigate the CEO-NE formulations (Hitachi High Technologies, USA). To visualize the nanodroplets’ outer surface and physical size, the samples were placed on a polycarbonate substrate, dried at room temperature, and then placed in a critical dryer. The samples were then coated with gold and examined [35]. Transmission electron microscopy was utilized to obtain data regarding the structure of CEO-NE (TEM) (JEM-100CXII, Hitachi Co. Ltd., Japan). Briefly, a droplet of CEO was placed on a copper wire with a carbon film and then stained with a 2 wt% solution of phosphotungstic acid for 2 min. The stained droplets were air-dried for 10 min and then analyzed using TEM at an accelerating voltage of 200 kV [36,37].

### 4.3. Size Distribution, Particle Size, and Zeta Potential

The dynamic light diffusion (DLS) approach was utilized in conjunction with the Zetasizer Nano ZS, model ZEN3500, to conduct size analysis on nanoemulsions (Malvern Instruments, UK). Both the preparation of the samples and their analysis were carried out. It took 10 microliters of the nanoemulsion and 990 microliters of ionized water to create 100-times dilution. The material was placed in a disposable plastic cuvette and analyzed in triplicate at 25 °C.

To assess the zeta potential of the nanoemulsion that was loaded with CEO, the Zetasizer Nano ZS was utilized. This was accomplished by using a one-of-a-kind mixed-mode measurement known as phase analysis light scattering. This measurement makes it possible to identify with a high level of precision both the average zeta potential and the zeta potential distribution (M3-PALS).

### 4.4. % Entrapment Efficiency (%EE) of CEO-NE

In order to determine the %EE of CEO-NE, we implemented a modified version of the procedure published in previous papers [37,38]. We began by taking 2 mL of the prepared CEO-NE and placing it in ultra-centrifugal filter tubes. The tubes were then centrifuged at 3000 rpm for 25–30 min at room temperature. During this process, any unentrapped drugs were forced out of the tubes. To calculate the %EE, we utilized the following formula:

(1) $$\%EE=\frac{\left(Total\;amount\;of\;CEO-Amount\;of\;CEO\;in\;supernatent\right)}{Total\;amount\;of\;CEO}\times 100$$

### 4.5. Drug Release and Kinetics Studies of CEO-NE

We conducted a thorough in vitro drug release study using the dialysis bag method in a phosphate-buffered saline (PBS) medium with a pH of 6.0 at a temperature of 32 ± 2 °C. The study was conducted under constant magnetic stirring for 24 h. We added 3 mL of the prepared CEO-NE and 3 mL of bare CEO to the donor compartment, and the entire process was conducted under constant magnetic stirring. At fixed intervals, we collected 3 mL samples and immediately replaced the PBS in the sink to maintain optimal conditions. The study was conducted in triplicate and the results were recorded as mean ± SD. We analyzed the kinetic data using DDSolver software and fitted the release data to various release kinetics models such as zero-order, first-order, Higuchi diffusion, and Korsmeyer–Peppas models. We interpreted the data based on the r^2^ values obtained.

### 4.6. In Vitro Anti-Oxidant Activity

Two distinct methods, DPPH radical scavenging and hydrogen peroxide radical scavenging, were utilized to evaluate the levels of antioxidant activity possessed by CEO, CEO-NE, and the standard.

#### 4.6.1. DPPH Radical Scavenging Activity

Preparation of DPPH reagent: 0.1 mM solution of DPPH was prepared in methanol

##### Preparation of sample/standard 

To perform the DPPH study, we prepared 1 mg/mL of CEO, CEO-NE, and standard methanol stock solution. For this purpose, 1 mg of CEO, CEO-NE, and standard were used with methanol to prepare 1 mg/mL stock solution. After transferring various volumes, (20–100 µg/mL) of CEO, CEO-NE, and standard from the stock solution into a set of test tubes, methanol was added to increase the total volume up to 1 mL. The absorbance was then measured at 517 nm after 30 min of incubation in the dark at room temperature using 2 mL of gently mixed 0.1 mM DPPH.

##### Preparation of control

As a control, 1 mL of methanol was mixed with 2 mL of 0.1 mM DPPH solution and incubated for 30 min at room temperature in the dark. The control was run against methanol (in the blank) at 517 nm [39].

The percentage antioxidant activity of the sample/standard was calculated using the following formula:

% Inhibition = [(Ab of control − Ab of sample/Ab of control × 100]

#### 4.6.2. Hydrogen Peroxide Radical (H_2_O_2_) Scavenging Activity

A solution (40 mM) in a phosphate buffer (pH 7.4) was prepared. The hydrogen peroxide concentration was determined spectrophotometrically at an absorbance of 230 nm in a spectrophotometer (Systronic, India). An H_2_O_2_ solution was mixed with CEO and CEO-NE samples dissolved in 20, 40, 60, 80, and 100 µg/mL in DMSO (40 mM). After 10 min, the absorbance of H_2_O_2_ was measured at 230 nm compared to a blank solution consisting of phosphate buffer but containing no H_2_O_2_ [40]. The percentage of antioxidant activity of samples and standards was calculated using the following formula:

% Inhibition = [(Ab of control − Ab of sample/Ab of control × 100]

### 4.7. Determination of Minimum Inhibition Concentration (MIC) and Minimum Bactericidal Concentration (MBC) 

#### 4.7.1. Preparation of Nutrient Media

A total of 1 liter of distilled water was used to dissolve 28 g of nutritional agar medium. Before beginning the sterilizing process, the pH of the media was measured. The media were sterilized in an autoclave for 15 min at 121 °C under 15 pounds of pressure. After the sterilizing process, the media were left to cool but were not allowed to become solid. The nutrient media were poured into plates and then positioned in the laminar airflow until the agar became firm.

#### 4.7.2. Antibacterial Activity 

The antibacterial activity of CEO and CEO-NE was evaluated by the excellent diffusion test using the bacteria Klebsiella pneumonia (*K. pneumonia*) (MTCC 8911). To perform this assay, we first distributed the culture of bacterial strains onto the culture medium (NAM). Then, 100 mg of test samples (CEO and CEO-NE) were collected with 10 mL of solvent (methanol and DMSO) to prepare a 10 mg/ml stock solution. Then, 10 mg of the standard (amoxicillin and ofloxacin) was taken with 10 mL of solvent (distilled water) to prepare a 1 mg/mL solution. Then, the inoculum was prepared; the test organisms were inoculated with 10 mL of nutrient broth. The bacterial suspension was standardized to 10^8^ CFU/mL bacteria and stored in the shaker. Then, 50 µL of the broth inoculum (at 10^8^ CFU/mL) was withdrawn using a micropipette and transferred to a fresh, sterile, solidified agar media plate [41]. The inoculum was spread evenly on the sterile agar plate using a sterile spatula to inoculate it. Four 6 mm holes were drilled into the inoculated medium using a clean cork cleaner.

Samples at four different concentrations (0.25%, 5%, 7.5%, and 10%) were placed in one well plate, while 50 µL of the standard drug was placed in another well plate. The samples were incubated at 37 °C for 18 to 24 h after being allowed to diffuse at room temperature for about 30 min. After incubation, a distinct zone corresponding to the antibacterial activity of the tested drugs was formed around the wells. It was found that it was possible to observe and quantify the zone of inhibition (ZOI) in millimeters. A ruler was placed on the back of the inverted Petri plate to measure the areas to the nearest millimeter. A non-reflective dark background served as a base for the Petri dish, positioned a few centimeters from the top. The diameters of the depression and the entire zone of inhibition, visible to the naked eye, were measured [42].

### 4.8. Anticancer Activity

Alveolar lung adenocarcinoma cell line A549 cells were grown in RPMI-1640 medium with 10% heat-inactivated FBS and 1% penicillin-streptomycin solution (10,000 units of penicillin and 10 mg of streptomycin in 0.9% NaCl), and 2 mM L-glutamine at 37 °C in a humidified atmosphere of 5% CO_2_ air at 37 °C. The cells were subcultured in 75 cm^2^ cell culture flasks. The medium was changed every 3 days. Cytotoxic activities of CEO and CEO-NE at concentrations ranging from 1.562 μM to 25 μM/mL were evaluated via MTT assay. The cells were seeded in 96-well plates at a density of 2 × 10^4^ cells per well and incubated until confluency was 90–95%. Each well was treated with 100 μL medium containing the desired concentrations of CEO and CEO-NE and incubated for 24 h. MTT working solution (5 mg/mL) in the volume of 20 μL was added to each well and incubated for another 4 h. At the end of incubation, the medium was carefully removed, and 200 μL DMSO was added. The optical density at 570 nm was then measured using a microplate reader. Gentle agitation in a gyro shaker improves dissolution. Sometimes, moving the tube up and down may be necessary to completely dissolve the MTT formation crystals, especially in dense cultures. Absorbance was measured using a spectrophotometer or ELISA reader at a wavelength of 570 nm. The percent cell viability was calculated using the following formula:

% cell viability = Abs of treated cells/Abs of Untreated cells × 100

The IC_50_ value was determined using a linear regression equation, i.e., Y = Mx + C.

Here, Y = 50, and M and C values were derived from the viability graph.

### 4.9. Short-Term Physical Stability

A study was conducted to examine how temperature affects the stability of CEO-NE over a short period of time. The research followed guidelines set by the International Conference on Harmonization (ICH, 2003) and we stored CEO-NE formulations at both 4 °C and room temperature for 0, 15, and 30 days. Then, samples were taken and analyzed to measure the particle size, PDI, ZP, and %DEE at each storage interval [43].

## Figures and Tables

**Table 1:** Drug release kinetics of CEO and CEO-NE.

Formulation	Zero Order	First Order	Higuchi	Kors–Peppas
CEO	0.598	0.719	0.950	0.953
CEO-NE	0.498	0.978	0.916	0.907

**Table 2:** Comparative IC_50_ study of ascorbic acid CEO and CEO-NE via hydrogen peroxide and DPPH radical scavenging assay.

Treatment Given	IC_50_ (μg/mL)
DPPH radical scavenging assay of ascorbic acid	10.42 ± 0.28
DPPH radical scavenging assay of C.A. oil	42.65 ± 1.7
DPPH radical scavenging assay of C.A. nanoemulsion	34.75 ± 1.54
Hydrogen peroxide assay of ascorbic acid	18.11 ± 0.7
Hydrogen peroxide radical scavenging assay of C.A. oil	83.21 ± 2.1
Hydrogen peroxide radical scavenging assay of C.A. nanoemulsion	70.89 ± 1.9

**Table 3:** Stability study of CEO-NE formulations.

Days	Temperature	%EE	Particle Size	PDI	ZP (mV)
0th	4 ℃	63.65 ± 3.182	221.8 ± 11.09	0.38 ± 0.019	−5.6 ± 0.28
	Room temp.	63.65 ± 3.182	221.7 ± 11.085	0.38 ± 0.019	−5.6 ± 0.28
15th	4 ℃	64.75 ± 3.237	221.7 ± 11.085	0.38 ± 0.019	−5.6 ± 0.28
	Room temp.	63.45 ± 3.172	222.3 ± 11.115	0.45 ± 0.0225	−5.9 ± 295
30th	4 ℃	66.23 ± 3.311	221.7 ± 11.085	0.38 ± 0.019	−5.7 ± 0.285
	Room temp.	59.87 ± 2.993	226.9 ± 11.345	0.51 ± 0.0255	−6.8 ± 0.34

## Data Availability

Not applicable
